# Subacute ruminal acidosis in high-producing dairy cows: an integrated diet–microbiota–epithelium–host framework for diagnosis and nutritional management

**DOI:** 10.1186/s40104-026-01472-1

**Published:** 2026-08-13

**Authors:** Xinyi Zhang, Jingyi Xu, Yiyang Sun, Shixi Liu, Shengjun Zhai, Ziyi Zhao, Junhu Yao, Shengru Wu

**Affiliations:** College of Animal Science and Technology, Northwest A&F University, Yangling, Shaanxi 712100 China

**Keywords:** Epithelia, Immunity, Microbiota, Rumen homeostasis, Subacute ruminal acidosis

## Abstract

High-producing dairy cows are commonly fed energy-dense diets to meet the nutrient demands of intensive milk production, but excessive rumen-degradable starch and insufficient physically effective fibre increase the risk of subacute ruminal acidosis (SARA). Rather than being defined only by low ruminal pH, SARA reflects a failure of acid-load adaptation, in which acid production exceeds the capacity for buffering, epithelial absorption, microbial adaptation, and host recovery. This review examines SARA within a diet–microbiota–epithelium–host framework, linking dietary acid-load pressure with microbial dysbiosis, epithelial barrier dysfunction, host inflammatory responses, diagnostic interpretation, and nutritional management. Particular emphasis is placed on interpreting ruminal pH as an indicator of acid-load exposure, integrating host-response and organ-level signals as supportive evidence for risk assessment, and matching nutritional strategies to disrupted points in acid-load control. By connecting mechanisms with diagnosis and nutritional decision-making, this review provides a structured basis for earlier SARA risk recognition and more targeted nutritional management in high-producing dairy cows.

## Introduction

To meet the energy demands of high-producing dairy cows in intensive production systems, energy-dense concentrate-rich diets are widely used. In grain-based formulations, these diets often increase the supply of rumen-degradable starch (RDS), which provides rapidly fermentable substrate for microbial fermentation and volatile fatty acid (VFA) production [1, 2]. However, when high-RDS supply is combined with insufficient physically effective fibre, unstable intake, or inadequate feeding management, ruminal acid load may exceed the capacity for buffering, absorption, and adaptation, increasing the risk of subacute ruminal acidosis (SARA) [3–6].

SARA has traditionally been associated with repeated or prolonged episodes of moderate ruminal pH depression. Nevertheless, it should not be interpreted as a disorder defined only by a single low-pH threshold. Reported pH cut-off values vary among studies because ruminal pH is affected by sampling site, sampling method, monitoring system, diet type, feeding time, and evaluation criterion [4, 7, 8]. In dairy cows, commonly used thresholds range from approximately pH 5.5 to 5.8, and SARA assessment often combines the selected threshold with the duration or area under the curve for which pH remains below that value [9–11]. Moreover, cows exposed to similar pH conditions may differ in feeding behaviour, epithelial adaptation, inflammatory responses, clinical signs, and production losses [4, 7, 12]. Thus, ruminal pH is better interpreted as a core indicator of acid-load exposure rather than as an absolute stand-alone diagnostic standard.

Although ruminal pH monitoring remains the most direct method for identifying acidotic risk, conventional approaches are invasive or potentially stressful and are poorly suited for repeated herd-level application [13]. More importantly, SARA reflects broader disturbances in rumen fermentation, epithelial function, microbial stability, and host responses. These disturbances may contribute to reduced feed intake, impaired fibre digestion, altered milk production or composition, and increased risk of extra-ruminal health problems [12, 14–16]. Therefore, SARA is more appropriately viewed as a progressive failure of acid-load adaptation than as a simple low-ruminal-pH event.

Previous reviews have provided important foundations for understanding SARA, including its physiological causes, diagnostic uncertainty, gut-health consequences, and prevention strategies [4, 7, 12, 17]. However, these topics have often been discussed as separate aspects of SARA. A framework that connects dietary acid-load dynamics with microbial dysbiosis, epithelial absorptive and barrier capacity, microbial translocation-associated inflammation, diagnostic interpretation, and nutritional decision-making remains insufficiently developed. As a result, it remains difficult to distinguish initiating factors from downstream consequences, to evaluate diagnostic indicators according to biological progression, or to translate monitoring results into practical nutritional interventions.

This review uses high-producing dairy cows in intensive production systems as the central interpretive context. Evidence from other ruminants and experimental models is cited only when it provides relevant mechanistic or comparative support. Accordingly, this review follows a mechanism-to-diagnosis-to-management logic. It first summarizes how dietary and feeding factors create acidogenic pressure, then discusses microbial, epithelial, inflammatory, and extra-ruminal responses as linked consequences of impaired rumen adaptation. Diagnostic and monitoring tools are evaluated according to biological relevance and practical applicability, and nutritional strategies are discussed as coordinated approaches for acid-load control and recurrence prevention. Through this structure, the review aims to support earlier SARA risk recognition and more precise nutritional regulation in high-producing dairy cows.

## Search strategy and evidence organization

This article was developed as a structured narrative review rather than a systematic review or meta-analysis. Relevant literature was identified through PubMed, Web of Science, and Google Scholar without a strict publication-year restriction. Recent studies were prioritized where they provided updated evidence on SARA mechanisms, diagnostic technologies, biomarkers, and nutritional management, whereas seminal older studies were retained when they provided foundational information on rumen physiology, ruminal pH thresholds, fibre effectiveness, buffering capacity, starch degradability, or established SARA concepts. Search terms included combinations related to SARA, ruminal pH, rumen-degradable starch, rapidly fermentable carbohydrate, physically effective fibre, rumen microbiota, epithelial barrier function, LPS, inflammation, diagnostic biomarkers, milk Fourier-transform infrared spectroscopy (FTIR), multiomics, buffers, yeast, and nutritional management. In vivo studies in lactating or high-producing dairy cows were treated as the primary evidence for diagnosis, production outcomes, feeding behaviour, nutritional management, and practical intervention. Dairy-cow in vitro, tissue, and epithelial cell studies were used as mechanistic support. Evidence from dairy goats, dairy sheep, lambs, beef cattle, other non-dairy ruminants, and non-cow in vitro systems was interpreted as comparative or hypothesis-generating evidence only.

## Pathogenesis and pathophysiology of SARA: from acid-load initiation to host response

Pathogenesis refers to the initiating events and causal sequence by which SARA develops, whereas pathophysiology refers to the functional disturbances that follow the destabilization of ruminal homeostasis. A clear mechanistic interpretation of SARA requires recognition that it differs fundamentally from acute ruminal acidosis in severity, fermentation pattern, clinical presentation, and adaptive failure. Acute ruminal acidosis is typically associated with a rapid and severe decline in ruminal pH, often below approximately 5.0, marked lactate accumulation, systemic metabolic acidosis, dehydration, and overt clinical disease [7, 18]. By contrast, SARA involves repeated or prolonged moderate pH depression as the measurable outcome of an imbalance among VFA production, salivary and dietary buffering, rumen mixing, epithelial absorption, and acid–base regulation [4, 17]. Within the integrated diet–microbiota–epithelium–host system, SARA can be interpreted as a sequential failure of acid-load adaptation. Dietary acid-load pressure first exceeds the rumen–host capacity for buffering, absorption, and adaptation, thereby destabilizing ruminal fermentation. This instability promotes microbial dysbiosis, epithelial dysfunction, and microbial translocation-associated inflammation, linking local ruminal disturbance with host inflammatory and metabolic stress. Accordingly, dietary acid-load pressure and fermentation instability are treated as initiating events, whereas microbial, epithelial, inflammatory, and organ-level responses are interpreted as consequences or modifiers of disease progression.

### Initiation of SARA: dietary acid-load pressure and fermentation instability

**Fig. 1:**
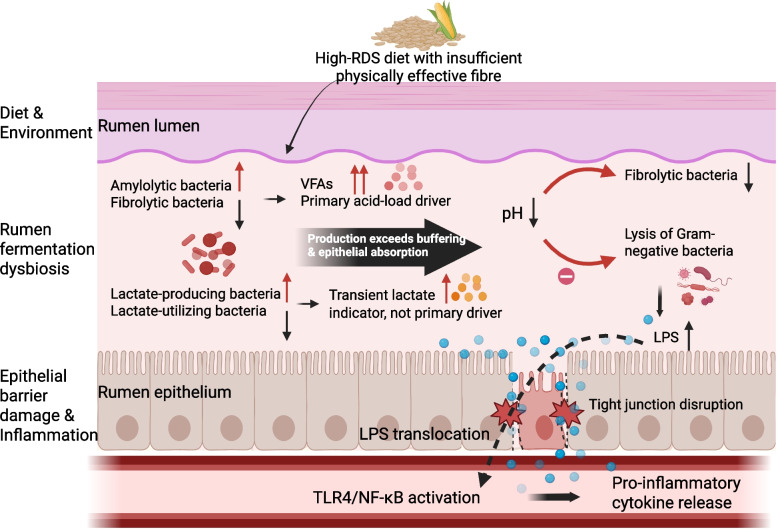
Schematic representation of ruminal fermentation dysbiosis, epithelial barrier disruption, and microbial translocation-associated inflammation during SARA. High-RDS diets with insufficient physically effective fibre promote amylolytic fermentation and increase VFA production. When VFA production exceeds ruminal buffering and epithelial absorption, ruminal pH declines, fibrolytic activity is suppressed, and microbial turnover or Gram-negative bacterial lysis may increase LPS release. Excessive acid load and microbially derived molecules impair epithelial barrier integrity, facilitating the translocation of LPS and other microbial products and activation of TLR4/NF-κB signalling, thereby promoting local rumen epithelial and systemic inflammatory responses. RDS, rumen-degradable starch; VFAs, volatile fatty acids; LPS, lipopolysaccharide; TLR4, toll-like receptor 4; NF-κB, nuclear factor-kappa B

The initiation of SARA reflects a mismatch between ruminal acid production and the rumen–host capacity for buffering, epithelial absorption, acid–base regulation, and microbial adaptation (Fig. 1). The rumen epithelium contributes to this balance by absorbing organic acids, particularly VFAs, and by regulating epithelial acid–base transport through mechanisms such as bicarbonate transport and Na⁺/H⁺ exchange [19]. High RDS supply from grain-based diets can favour amylolytic and acid-tolerant bacteria, accelerate starch fermentation, and increase VFA production [20–22]. Under physiological conditions, VFAs are absorbed across the rumen epithelium; however, during SARA, excessive VFA flux increases the demand for epithelial acid–base regulation and intracellular pH homeostasis, which may contribute to epithelial stress and barrier dysfunction [19, 23]. Although reduced ruminal pH can disturb the balance between lactate-producing and lactate-utilizing bacteria, typical SARA is generally driven more by VFA accumulation and inadequate buffering or epithelial absorption than by persistent lactate accumulation [12, 19, 24–26]. Physically effective fibre is therefore essential for ruminal acid–base stability because it promotes chewing-associated salivary buffering, supports rumen mat formation, and moderates substrate fermentation rate [27, 28]. In contrast, insufficient effective fibre or prolonged low pH reduces acid-buffering capacity, suppresses fibrolytic activity, decreases fibre digestibility, and contributes to ruminal instability [12, 29–33]. Overall, SARA progression is driven by the accumulation of VFAs and other acidic microbial metabolites, together with altered ruminal pH dynamics and insufficient adaptive capacity [34–36].

### Functional mechanisms: microbial dysbiosis, epithelial dysfunction, and microbial translocation-associated inflammation

Microbial responses during SARA should be interpreted primarily as functional shifts rather than as fixed taxonomic signatures. Under acidogenic dietary conditions, especially when high-RDS or starch-dominant diets are combined with insufficient effective fibre, rumen fermentation tends to shift from fibrolytic and fibre-associated functions toward rapidly fermentative, starch-utilizing, acidogenic, and endotoxin-related functions [4, 37–39]. At the phylum level, concentrate-rich or starch-dominant diets have produced variable shifts in Firmicutes and Bacteroidetes across studies [34, 37–40]. Members of Bacteroidetes in the dairy-cow rumen encode diverse carbohydrate-active enzymes involved in the degradation of plant structural carbohydrates [41]. However, genus-level responses are more variable because they are affected by host species, diet formulation, carbohydrate structure, sampling site, feeding behaviour, and sequencing method. Taxa such as *Prevotella*, *Selenomonas*, *Ruminococcus*, *Bifidobacterium*, and *Mitsuokella* have been reported in different SARA-related models, but these taxa should be interpreted as context-dependent indicators rather than universal biomarkers of SARA or SARA tolerance [3, 42–48]. Therefore, Table 1 summarizes microbial responses according to production type, dietary model, carbohydrate structure, analytical method, and direction of change. Dairy-cow studies are used for cow-focused interpretation, whereas dairy-goat evidence is retained as comparative mechanistic support. These context-dependent responses may be further shaped by feed sorting behaviour, host adaptive capacity, and the type of fermentable or fibre substrate available for ruminal fermentation [54–57].

**Table 1:** SARA-related rumen microbial responses in lactating dairy cows and comparative goat models

Production type	Dietary model	Dominant carbohydrate structure	Decreased taxa	Increased taxa	Analytical method	Reference
Goat model	Increasing dietary grain/HG challenge	Not fully specified	Bacteroidetes, Fibrobacteres	Firmicutes	16S rRNA V3–V4; Illumina HiSeq	[48]
Lactating Holstein dairy cows	HG diet	60% concentrate diet; corn-based high-grain/starch supply	*Methanobrevibacter*, Lachnospiraceae ND3007_group	*Prevotella*, *Bacteroides*; *Stenotrophomonas*, and Xanthomonadaceae	16S rRNA V3–V4; Illumina NovaSeq/HiSeq Xten	[49]
Dairy goats	HC diet with whole-corn and crushed-corn models	Whole-corn and crushed-corn models used to modify starch degradability	*Prevotella*, *B. adolescentis*	Firmicutes, Proteobacteria, *Ruminococcus*	Metagenomic sequencing; Illumina NovaSeq	[42]
Dairy goats	HC, corn-based starch model	70% concentrate diets supplemented with whole corn or crushed corn	*Prevotella*, Bacteroidales	*Ruminococcus*, *Selenomonas*, *Bifidobacterium*, *Mitsuokella*	Metagenomic sequencing; Illumina NovaSeq and HiSeq Xten	[3]
Dairy cows	Same SARA-inducing diet used to classify susceptible and tolerant cows	Not fully specified	*Ruminococcaceae* spp., *Ruminococcus* spp., *Papillibacter*	*Prevotella* spp.	16S rRNA V3–V4; Illumina MiSeq	[50]
Lactating dairy cows	HG diet	60% concentrate; grain/starch	Bacteroidota, Spirochaetota, Fibrobacterota	Proteobacteria	16S rRNA V3–V4; Illumina MiSeq	[51]
Lactating Holstein cows	SARA induction model	Basal diet plus 10% wheat/barley pellet	Firmicutes	Bacteroidetes, *Prevotella*, YRC22; *Prevotella*, *Ruminococcus*, *Streptococcus*, and *Lactobacillus*	16S rRNA paired-end sequencing; Illumina MiSeq; qPCR	[52]
Lactating dairy cows	HG diet	60% concentrate; grain/starch	Bacteroidetes, *Bacteroides*, *Fibrobacter*, *Alistipes*, Candidatus Methanoplasma, *Methanomassiliicoccus*, *Methanolobus*	*Ruminococcus*, *Eubacterium*, *Selenomonas*, *Ruminobacter*, *Succinimonas*, *Methanomicrobium*, *Methanocaldococcus*	Metagenomic sequencing; Illumina HiSeq Xten	[53]

*SARA* Subacute ruminal acidosis, *HC* High-concentrate, *HG* High-grain, *TMR* Total mixed ration

Protozoal shifts should be considered modulatory in SARA. Protozoa may influence starch sequestration, bacterial predation, nitrogen recycling, and fibre-associated digestion, thereby modifying fermentation kinetics under acidogenic diets [58–65]. However, current evidence for protozoal responses is less consistent than that for dietary acid-load pressure, microbial functional dysbiosis, epithelial barrier impairment, and LPS-related inflammatory responses. Therefore, protozoal changes are discussed here as context-dependent modifiers whose importance may vary with diet, host species, sampling site, and experimental model.

Epithelial dysfunction represents a key step by which ruminal fermentation disturbance may progress toward host-level inflammatory stress. Under sustained acid-load pressure, the rumen epithelium faces increased demand for VFA absorption, epithelial acid–base regulation, and barrier maintenance to limit acid accumulation and microbial translocation. Abrupt transitions to more fermentable concentrate-rich diets, particularly in controlled challenge models using diets containing more than 60% concentrate on a dry matter (DM) basis, may provide insufficient time for epithelial adaptation to increased VFA flux and low-pH exposure [66, 67]. When epithelial transport capacity or barrier integrity is impaired, microbial-derived products and inflammatory mediators may translocate more readily across the rumen epithelium, providing a pathway through which local ruminal dysbiosis may contribute to systemic inflammatory responses [68].

Biogenic amines may further modify this process by affecting rumen motility, epithelial integrity, and inflammatory responses. Among biogenic amines, histamine has been associated with reduced feed intake and rumen motility [69]. Experimental evidence also suggests that histamine may aggravate inflammatory responses and impair rumen epithelial cell growth, maturation, and permeability regulation [70]. Elevated VFA concentrations, particularly under acidotic pH conditions, may further reduce tight junction integrity and compromise epithelial structure [71–73].

Among immune-mediated processes, LPS-related inflammatory signalling provides the clearest link between ruminal microbial dysbiosis, epithelial barrier impairment, and systemic host responses. Sustained acid-load pressure may increase microbial turnover and LPS release, while epithelial barrier disruption facilitates the translocation of microbial products into portal or systemic circulation. These signals can activate Toll-like receptor 4 (TLR4)/NF-κB-related inflammatory pathways and hepatic acute-phase responses, as reflected by host-response markers such as LPS-binding protein (LBP), serum amyloid A (SAA) and haptoglobin (Hp) [74–79]. At the organ level, the liver acts as a major immunometabolic filter for rumen-derived inflammatory molecules, whereas distal epithelial tissues such as the mammary gland may also be affected when circulating microbial products or cytokines interact with local barriers [80–83]. Compared with individual microbial taxa, protozoal shifts, or biogenic amines, this pathway provides a more direct basis for connecting ruminal disturbance with host inflammatory stress. Other factors should therefore be interpreted as modulators or amplifiers whose importance may vary with diet, species, sampling site, and experimental model.

### Clinical manifestations and systemic disease associations of SARA

The systemic relevance of SARA should be interpreted according to the strength of evidence for each outcome. Production changes and ruminal fermentation disturbances are relatively well supported in dairy cows, whereas systemic inflammation and extra-ruminal organ effects are supported by a combination of dairy-cow challenge studies, mechanistic evidence, and comparative models.

#### Reduced milk fat percentage

Reduced milk fat percentage is a practical herd-level warning indicator that has been associated with SARA, but it should not be interpreted as a universal or SARA-specific clinical symptom. In early lactation multiparous Holstein cows with ruminal acidosis, both fat-corrected milk yield and milk fat yield decreased [16]. Mechanistically, sustained ruminal pH depression may suppress fibrolytic activity, alter VFA profiles, reduce acetate availability, and shift rumen biohydrogenation toward milk fat-inhibitory intermediates [84–87]. However, milk fat depression is also affected by dietary fat, fibre source, starch level, lactation stage, breed, season, and other metabolic or nutritional disorders. Therefore, milk fat percentage is best used as a herd-level screening signal of possible fermentation instability and should be interpreted together with ration structure, ruminal pH dynamics, feeding behaviour, and production records.

#### Intestinal dysfunction

SARA-associated intestinal dysfunction is mainly related to the amount and type of fermentable substrate escaping ruminal digestion and entering the hindgut. In dairy cows, SARA challenge can alter microbial richness and diversity across the gastrointestinal tract, especially in the rumen and large intestine, suggesting reduced cross-site microbial stability [88]. Grain-based SARA appears to have stronger effects on cecal and fecal bacterial communities than alfalfa-pellet-induced SARA, indicating that hindgut dysbiosis is shaped not only by low pH but also by the type of fermentable substrate reaching the lower gut [89].

This substrate-driven microbial shift may increase endotoxin load and epithelial injury. In dairy cows, grain-based SARA increased free LPS in rumen fluid and cecal digesta and was associated with greater starch flow to the cecum [89]. Comparative dairy-goat evidence further supports a conserved hindgut-injury mechanism under acidogenic feeding, but these findings should not be interpreted as direct evidence for the magnitude or prevalence of hindgut dysfunction in high-producing dairy cows [90, 91].

#### Lameness and laminitis

Hoof disorders, including laminitis and lameness, have been repeatedly associated with SARA, but the causal pathway remains incompletely resolved. Current evidence supports a biologically plausible link between acidotic challenge, inflammatory mediators, vascular disturbance, and hoof lesions, although not all claw lesions respond uniformly to SARA severity [92]. Therefore, lameness should be interpreted as a potential downstream health outcome rather than as a specific diagnostic marker of SARA.

#### Liver abscess

Liver abscess represents a biologically plausible but incompletely established extra-ruminal consequence of gastrointestinal barrier disruption in dairy cows. Most direct evidence for the classical rumenitis–liver abscess complex derives from feedlot cattle, whereas its prevalence and causal relationship with SARA in high-producing dairy cows remain insufficiently defined [93, 94]. Beyond abscess formation, SARA-associated LPS and histamine exposure may induce hepatic inflammatory injury, indicating that the liver should be viewed as an immunometabolic target organ rather than only as a site of bacterial emboli [95].

#### Mammary inflammation

Mammary dysfunction during SARA should be interpreted as a possible downstream outcome of rumen or intestinal barrier disruption rather than as a SARA-specific consequence. Barrier disruption may allow microbial-derived molecules, including LPS, histamine, and peptidoglycan-derived molecules such as γ-D-glutamyl-meso-diaminopimelic acid (iE-DAP), to reach the mammary gland and activate inflammatory pathways [96–102]. These responses may impair blood–milk barrier integrity and mammary epithelial function. However, mammary inflammation is influenced by many local and systemic factors, and current evidence should be interpreted as supportive of a rumen/gut–mammary inflammatory link rather than as proof of a universal SARA outcome.

#### Reproductive tract inflammation

Reproductive tract inflammation may occur as a downstream response to digestive tract-derived inflammatory signals during SARA, but the current evidence remains limited. In mid-lactating dairy cows, digestive tract-derived LPS during SARA has been linked with uterine inflammatory signalling [99]. Long-term high-concentrate feeding decreased ruminal pH, increased ruminal and circulating LPS levels, and activated uterine TLR4–NF-κB inflammatory signalling. Consistently, bovine endometrial cell studies show that LPS can induce TLR4/myeloid differentiation factor 88 (MyD88)-dependent NF-κB activation and cytokine responses [103]. Therefore, reproductive tract inflammation should be interpreted as a possible systemic consequence requiring further validation rather than as a primary diagnostic feature of SARA.

## Nutritional determinants, susceptibility, and management strategies for SARA

Because SARA reflects a mismatch between acid production and the rumen–host capacity for buffering, epithelial absorption, microbial adaptation, and recovery, nutritional management should first target the basal ration and feeding behaviour. In high-producing dairy cows, the primary priorities are to control starch/RDS supply, maintain adequate peNDF, regulate grain processing intensity, improve TMR uniformity, reduce feed sorting, manage feeding frequency, ensure consistent feed allowance, and support gradual dietary transition. Functional additives, including buffers, yeast-based products, butyrate-related epithelial support, antioxidants, thiamine, enzymes, or phytogenic compounds, should be positioned as adjunctive tools rather than replacements for correction of the underlying acid-load imbalance. Therefore, nutritional risk factors, individual susceptibility, and management strategies are interpreted here as interconnected components of the same acid-load management framework.

### Dietary acid-load pressure and effective fibre balance

Feed composition and formulation are central determinants of SARA risk because they influence the balance between fermentable substrate supply and ruminal buffering capacity. Prediction approaches, including near-infrared spectroscopy-based models, can help evaluate feed characteristics related to acidogenic potential, such as starch availability, fibre effectiveness, and raw material properties [104]. Diets rich in rapidly fermentable carbohydrates increase the rate of VFA production, whereas insufficient effective fibre weakens the rumen’s capacity to distribute, buffer, and clear postprandial acid load [105–107]. Therefore, concentrate proportion should be interpreted together with fibre effectiveness, starch degradability, dry matter intake (DMI), and feeding management [84, 108]. In controlled challenge models, SARA has frequently been induced using high-concentrate diets containing 60% or more concentrate [109]. In one controlled study, increasing the forage-to-concentrate ratio from 35:65 to 60:40 on a DM basis increased mean ruminal pH and reduced the daily time below pH 5.8 [110]. Nevertheless, this specific dietary comparison should not be interpreted as establishing a universal concentrate target for high-producing dairy cows. Moreover, diets with similar concentrate levels may differ substantially in starch source, RDS content, particle size, forage effectiveness, buffering capacity, feed sorting potential, and intake pattern. Thus, the practical target is not simply to lower the concentrate proportion, but to balance fermentable substrate supply with physically effective fibre and epithelial acid-clearance capacity.

Fibre-related risk depends on both chemical fermentability and physical effectiveness. Potentially fermentable neutral detergent fibre (pfNDF), mainly derived from cellulose and hemicellulose fractions, supports fibrolytic fermentation and provides a slower release of fermentable substrates [111, 112]. Physically effective neutral detergent fibre (peNDF) modifies acid-load risk by stimulating chewing, rumination, saliva secretion, rumen mat formation, rumen motility, and digesta passage [112, 113]. Thus, insufficient peNDF is particularly problematic when dietary starch or RDS supply is high [114]. Available quantitative recommendations support the need to evaluate starch and peNDF together. In dairy cows, models suggest approximately 31.2% peNDF retained above 1.18 mm or 18.5% peNDF retained above 8 mm on a DM basis [115]. A practical range of 15%–18% peNDF > 8 mm has also been proposed when dietary starch is maintained at approximately 20%–25% of DM or non-fibre carbohydrates at 35%–40% of DM [5]. In mid-lactation Holstein cows fed a high-concentrate diet, increasing dietary peNDF8.0 by reducing total mixed ration mixing time increased chewing and rumination activity, elevated ruminal pH, increased acetate proportion and the acetate-to-propionate ratio, and reduced propionate proportion. These findings indicate that peNDF does not simply represent a minimum fibre target, but functions as a physical modifier of fermentation pattern and acid-load distribution under high-concentrate feeding conditions [116].

Starch source and processing intensity further determine the acidogenic potential of a diet. Highly fermentable grain sources and intensive grain processing can increase ruminal starch availability and accelerate fermentation. Barley generally has a faster ruminal starch fermentation rate than corn, and finely processed corn can increase the surface area available for microbial attachment and ruminal starch digestion [117–119]. By contrast, some non-forage fibre sources, such as soy hulls or distillers’ grains, may partially replace cereal grains and reduce the dietary load of rapidly fermentable starch, although responses depend on fibre source, inclusion level, and basal diet composition [120, 121].

Acid-load risk is not restricted to indoor high-concentrate feeding systems. Grazing cows may also experience low ruminal pH when highly digestible pasture contains abundant water-soluble carbohydrates but insufficient physically effective fibre [122]. This risk may be particularly relevant in spring, when temperate grasses can contain high levels of rapidly fermentable sugars and fructans, and fructans can be rapidly catabolized by bovine rumen microbiota [123, 124]. Low ruminal pH has also been reported in grazing dairy cows consuming perennial ryegrass-based pasture, and substitution of grain with fructose has been shown in a specific sugar-based challenge to decrease ruminal pH and increase VFA concentrations [125]. However, pasture-associated low-pH conditions may differ from grain-based SARA in microbial, inflammatory, and epithelial outcomes [4]. Therefore, grazing-related SARA risk should be interpreted according to pasture maturity, water-soluble carbohydrate content, effective fibre supply, concentrate supplementation, and grazing management.

Overall, dietary acid-load control requires coordinated management of rapidly fermentable carbohydrate supply, starch/RDS degradability, peNDF adequacy, and ruminal buffering and absorption capacity. The central principle is to prevent a mismatch between acid production and the rumen’s capacity to buffer, absorb, and clear fermentation acids.

### Feed processing, TMR management, and dietary transition

Feed processing and TMR management influence SARA risk by changing the rate and uniformity of fermentable substrate delivery. Evidence from lamb models indicates that pelleted high-grain TMR can alter ruminal fermentation and epithelial responses [126, 127]; however, these studies are used here only to illustrate the general principle that feed physical form can modify acid-load delivery. When pelleted TMR contains high levels of concentrate or grain, pelleting may reduce particle size, decrease peNDF, increase starch availability, and accelerate fermentation, thereby increasing acid-load pressure [128, 129].

Starch processing should be managed according to the ruminal degradation rate. Slowly fermentable starch sources or combinations of starch sources may moderate fermentation kinetics, whereas excessive grinding, fine processing, or gelatinization should be avoided when acid-load risk is high [130]. Comparative evidence from dairy goats shows that, compared with ground corn, whole corn kernels can lower ruminal starch degradation rate and reduce inflammatory or diarrhoeal responses [130]. These findings are consistent with the cow-focused principle that starch delivery rate, rather than starch content alone, contributes to acid-load risk.

The technical value of TMR lies in reducing variation between the formulated diet and the consumed diet. A well-managed TMR can homogenize nutrient intake, reduce selective consumption of starch-rich or low-fibre fractions, and limit abrupt postprandial changes in ruminal fermentation. Forage particle length should be sufficient to maintain physically effective fibre, stimulate chewing activity, support rumen mat formation, and reduce feed sorting, although the optimal particle size depends on forage source, moisture, mixing procedure, and overall ration composition [131, 132]. Stable feed delivery is equally important: inconsistent feed allowance, prolonged empty-bunk periods, irregular delivery, insufficient feed push-up, or excessive feed competition may promote erratic intake and large meals, increasing short-term acid-load peaks even when the formulated ration is appropriate. Therefore, consistent feed allowance, regular TMR delivery and push-up, adequate refusals, and reduced feed competition are practical measures to stabilize substrate intake.

Dietary transition is another critical point in SARA prevention and should be managed as a stepwise increase in fermentable substrate supply rather than an abrupt dietary shift. Depending on the extent of dietary change and microbial group, rumen microbial adaptation may require several days to more than 3 weeks [38, 133]. Therefore, RDS supply should be increased progressively while maintaining adequate peNDF, monitoring DMI and sorting behaviour, and avoiding sudden changes in grain source or processing intensity.

Overall, feed processing, TMR management, and dietary transition should be understood as management tools that regulate the rate, physical form, and temporal pattern of substrate delivery. Their goal is not only to supply sufficient energy, but also to prevent rapid postprandial acid-load peaks by coordinating starch degradation rate, effective fibre structure, feed uniformity, microbial adaptation, and epithelial absorptive capacity.

### Physiological stage, feeding behaviour, and individual susceptibility

Physiological stage and individual feeding behaviour further influence SARA susceptibility. Transition and early-lactation cows are particularly vulnerable because DMI often decreases around calving or recovers more slowly than the rapid increase in nutrient demand after lactation begins [134–137]. This mismatch may encourage rapid increases in dietary concentrate supply after calving [135, 138–140]. This combination of high nutritional demand, changing ration structure, unstable intake, and variable feeding behaviour makes this stage particularly susceptible to ruminal acid-load instability.

**Fig. 2:**
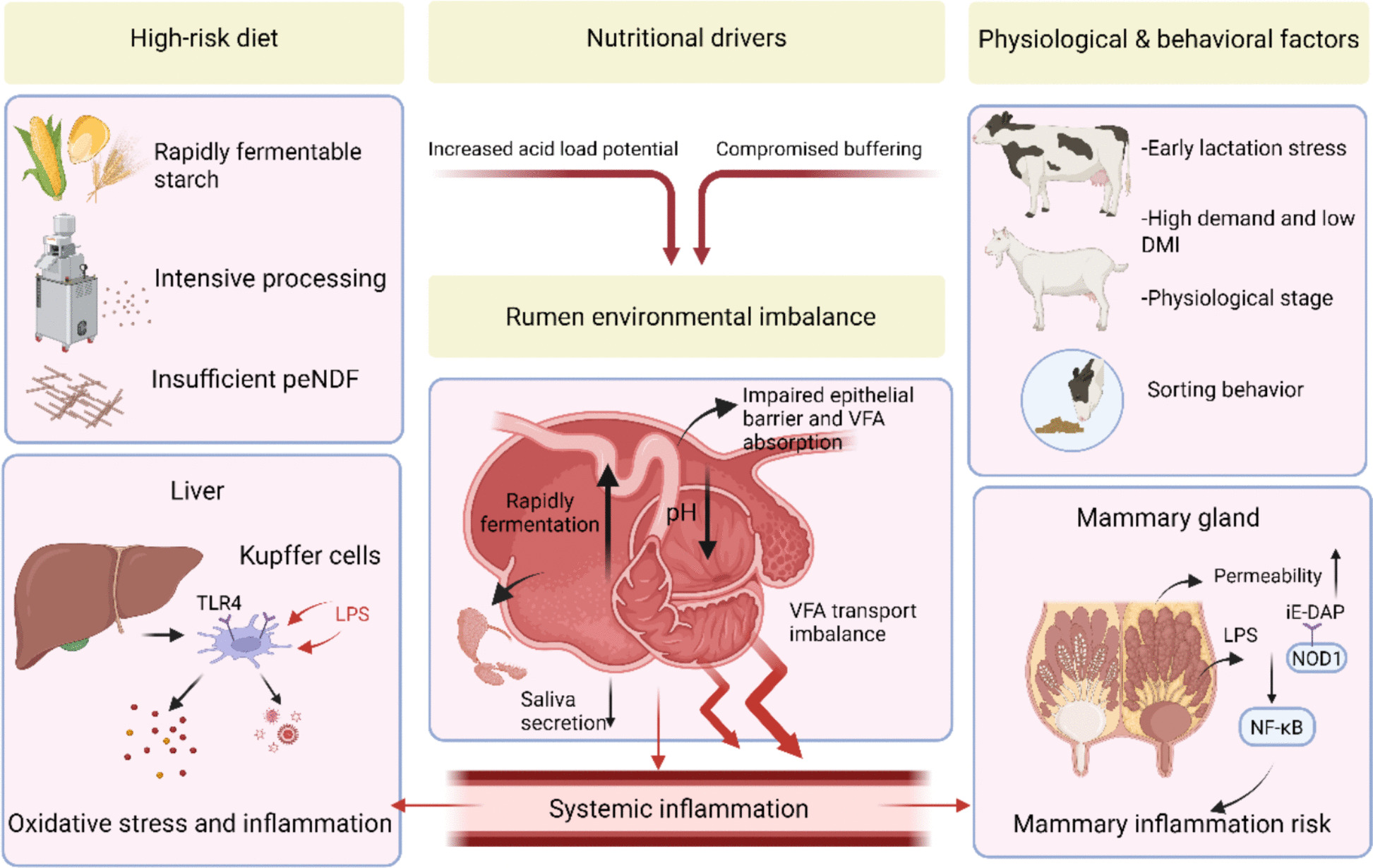
Nutritional and host-related drivers linking ruminal acid-load imbalance with systemic inflammatory responses during SARA. High-risk diets rich in rapidly fermentable starch and low in physically effective fibre increase ruminal acid load, while reduced chewing and salivary buffering limit pH control. This promotes rumen fermentation imbalance, pH depression, and impaired epithelial barrier function, facilitating the translocation of microbial-derived inflammatory molecules into circulation. Systemic effects include liver-mediated inflammatory responses and increased risk of mammary gland inflammation. Physiological and behavioural factors, such as early lactation stress, low feed intake, and feed sorting, further modulate individual susceptibility to SARA. SARA, subacute ruminal acidosis; peNDF, physically effective neutral detergent fibre; VFA, volatile fatty acid; LPS, lipopolysaccharide; TLR4, Toll-like receptor 4; DMI, dry matter intake; iE-DAP, γ-D-glutamyl-meso-diaminopimelic acid; NOD1, nucleotide-binding oligomerization domain-containing protein 1; NF-κB, nuclear factor kappa B

Individual feeding behaviour can create different acid-load exposures even under the same formulated diet. Increasing feeding frequency may reduce postprandial fluctuations in ruminal pH by distributing intake more evenly throughout the day [141]. However, this effect depends on meal size, eating rate, sorting behaviour, ration structure, and effective fibre intake. Cows that consume large meals, eat rapidly, or selectively consume starch-rich and low-fibre fractions may still experience high short-term acid loads, particularly when diets are rich in rapidly fermentable carbohydrates or prone to feed sorting [2, 54]. In silage-based diets, preformed organic acids may also contribute to acid exposure, but the extent of ruminal pH depression is largely shaped by DMI, rumination, sorting behaviour, and physically effective fibre intake [142, 143]. Thus, feeding behaviour should be considered an individual-level modifier of acid-load exposure. Furthermore, cows may differ in epithelial VFA transport and barrier resilience, hepatic detoxification capacity, inflammatory responsiveness, and recovery after acidotic challenge. For example, the liver acts as a major immunometabolic filter for rumen- and intestine-derived inflammatory molecules, whereas the mammary gland must balance milk component synthesis, immune defence, and epithelial barrier maintenance under altered nutrient and inflammatory conditions [81, 86, 98, 144, 145] (Fig. 2). These organ-level responses should not be interpreted as primary causes of SARA, but as factors that may influence whether ruminal acid-load imbalance progresses to systemic inflammation.

Together, these findings provide a rationale for future precision-feeding frameworks that consider transition status, feed-sorting behaviour, ruminal pH recovery, epithelial barrier indicators, and inflammatory responses. However, susceptibility classification based on epithelial, hepatic, or immune resilience remains insufficiently defined.

## Functional additives as adjunctive preventive strategies

Functional additives should be positioned as adjunctive tools rather than substitutes for appropriate diet formulation and feeding management. Their practical value depends on the basal diet, acid-load severity, physiological stage, supplementation dose, and target mechanism. In general, buffers mainly target ruminal buffering capacity; yeast-based products mainly target microbial fermentation stability, endotoxin burden, and epithelial resilience; and other functional additives may support fibre degradation, fermentation kinetics, oxidative balance, or inflammatory regulation.

### Buffers

The primary role of buffers is to increase ruminal buffering or alkalinizing capacity and reduce the duration or severity of postprandial pH depression when acid load is high. The natural buffering capacity of the rumen depends partly on saliva production and feed characteristics [146]. Common buffering or alkalinizing agents include sodium bicarbonate, magnesium oxide, calcium carbonate, and alkaline mineral complexes [147–149]. These compounds can help neutralize acidic metabolites and stabilize the ruminal environment, but their effects on DMI, milk yield, and milk fat content are not always consistent [150]. Their efficacy depends on supplementation dose, basal diet composition, physiological stage, and the severity of ruminal acid load (Table 2).

**Table 2:** Buffering and alkalinizing strategies evaluated in dairy cows and comparative models

Buffering agent or formulation	Animal	Stage of supplementation	Addition level	Effect	Reference
Encapsulated sodium bicarbonate, calcium carbonate, magnesium oxide	Dairy cows	Late-lactation cows	200 g/d, 125 g/d or 75 g/d	Ruminal pH showed no significant change	[151]
Alkaline mineral complex (including potassium bicarbonate and sodium metasilicate pentahydrate)	In vitro rumen fermentation model	Not applicable	1, 2, 4, and 8 mL/kg of the substrate	Increased ruminal pH	[152]
Sodium butyrate, sodium bicarbonate	Goats	Lactating	10 g sodium butyrate and 10 g sodium bicarbonate per goat	Increased milk fat yield	[153]
Alkaline mineral complex buffer (including sodium metasilicate, potassium bicarbonate, zinc oxide)	Dairy cows	Early-lactation	10 g sodium metasilicate, 5 g potassium bicarbonate, and 0.5 mg zinc oxide per cow	Increased dry matter intake, milk production, fat-corrected milk yield, energy-corrected milk yield, lactose and milk protein concentrations, and daily yields	[147]
Sodium bicarbonate	Dairy cows	Lactating	2.5 g/L water	No significant change	[154]

Evidence from goats and in vitro systems was used only as comparative or mechanistic support

### Yeast-based products, probiotics, and candidate microbial targets

Yeast-based products are better positioned as preventive or early risk-reduction tools rather than primary treatments after SARA has developed. Compared with many other functional additives, yeast-based products have been more frequently evaluated in dairy-cow SARA-related studies and may support rumen fermentation stability and host-response modulation (Table 3). Active dry *S. cerevisiae* has been reported to improve rumen function, increase cellulolytic microorganisms, raise ruminal pH, and increase the abundance of the lactate-utilizing bacterium *Megasphaera elsdenii* during SARA challenge [156, 157]. *S. cerevisiae* fermentation products have also been associated with reduced ruminal or peripheral endotoxin concentrations, lower inflammatory markers, enrichment of fibrolytic taxa, and stabilization of microbial network structure during grain-based SARA challenge [158, 159, 162].

**Table 3:** Evaluated microbial interventions and exploratory microbial or metabolite targets in SARA-related models

Microbial product, strain, or candidate intervention	SARA-related model	Main outcomes	Reference
Yeast β-glucan	SARA-related yak rumen epithelial cell model	Regulated cell growth, metabolism, and protein synthesis	[155]
Active dry *Saccharomyces cerevisiae* strain 1242	Abrupt transition from high-forage to HG diet in lactating dairy cows	Alleviated SARA, improved rumen function, and increased cellulolytic microorganisms	[156]
Active dry yeast, *Saccharomyces cerevisiae* Yea-Sacc 1026	HG diet in dairy cows	Tended to increase minimum ruminal pH, reduce time below pH 5.6 or 5.8, and increase *Megasphaera elsdenii* abundance	[157]
SCFP	HG diet in dairy cows	Reduced ruminal and peripheral endotoxin concentrations and inflammatory markers, including LPS, LTA, SAA, and IL-1β	[158]
SCFP	HG diet in dairy cows	Stabilized rumen liquid microbiota, promoted lactate-utilizing and fibrolytic bacteria, and improved microbial robustness	[159]
*Megasphaera elsdenii* NCIMB 41125	HC diet in early-lactation dairy cows	Reduced daily variation in time below pH 5.6 and shifted fermentation toward propionate	[160]
*Enterococcus faecium* alone or with *Saccharomyces cerevisiae*/*Lactococcus lactis*	Induced SARA challenge in rumen-fistulated Holstein cows	Combination treatments showed beneficial effects on pH regulation in induced SARA models	[161]

*SARA* Subacute ruminal acidosis, *HC* High-concentrate, *HG* High-grain, *SCFP Saccharomyces cerevisiae* fermentation products, *LTA* Lipoteichoic acid, *SAA* Serum amyloid A, *LPS* Lipopolysaccharide, *VFA* Volatile fatty acid

The benefits of live yeast may also extend to the transition period. Studies by Bach and colleagues showed that live *S. cerevisiae* supplementation during the transition period modulated rumen and colon microbiota and affected epithelial gene expression related to inflammation, barrier regulation, and antimicrobial defence [163, 164]. Therefore, yeast-based products should be interpreted as microbial and epithelial modulators that improve fermentation resilience, reduce endotoxin burden, and support host barrier and inflammatory homeostasis.

Beyond yeast-based products, several bacterial taxa and microbial metabolites have been associated with SARA tolerance or epithelial protection in goat or other mechanistic SARA-related models, but their use as probiotic interventions remains exploratory. *Bifidobacterium* and tryptophan-derived metabolites such as indole-3-acetic acid may participate in immune regulation and epithelial protection [3, 42]. However, *Bifidobacterium* spp. should be interpreted cautiously in the rumen because members of this genus can produce lactate. Under specific acidogenic substrate conditions, rapidly fermentable carbohydrates may promote D-lactate accumulation, increase *Bifidobacterium* and *Lactobacillus*, and reduce fibrolytic bacteria, suggesting that excessive enrichment of lactate-producing taxa may aggravate ruminal dysbiosis [159]. Therefore, these taxa should currently be regarded as candidate functional targets rather than validated probiotic strategies for SARA.

### Other functional additives

Beyond buffers and yeast-based products, other functional additives may provide supportive benefits under acidogenic feeding conditions, but their effects are generally more diet- and dose-dependent. Exogenous fibrolytic or amylolytic enzymes may improve fibre or starch utilization and alter ruminal fermentation, although dairy-cow responses are inconsistent and depend on enzyme type, forage source, concentrate level, and application method [165, 166]. Selected phytogenic compounds or essential-oil blends may modulate rumen fermentation, feeding behaviour, SARA risk, or systemic inflammatory markers, but their effects vary substantially with active compounds, dosage, and physiological stage [167–169]. Thiamine is more directly relevant under high-grain challenge conditions, because SARA can disturb thiamine status and thiamine supplementation may reduce ruminal lactate accumulation, improve pyruvate metabolism, and partially stabilize ruminal metabolic profiles [170, 171]. Antioxidant nutrients such as vitamin E may alleviate oxidative and inflammatory responses during high-grain-induced SARA and partly improve intake or milk fat-related responses [172]. Overall, these additives should be presented as supportive or experimental tools rather than primary prevention strategies for SARA.

### Staged recovery after SARA and recurrence prevention

Once SARA has been identified, nutritional correction should follow a staged recovery strategy. The first stage is to reduce ruminal acid-load pressure and restore the physical conditions required for stable fermentation. This can be achieved by temporarily reducing rapidly fermentable starch or RDS supply and increasing high-quality long-forage intake [173]. Buffering agents may also be used as supportive tools [174, 175]. Other supportive interventions, including selected phytogenic compounds, thiamine, and antioxidant nutrients, may help modulate fermentation, reduce oxidative stress, or support epithelial barrier recovery under acidotic or inflammatory conditions [170, 172, 176, 177]. However, these additives should be interpreted as secondary recovery-supporting tools rather than primary treatments for SARA.

After ruminal pH stabilizes or clinical signs improve, recovery management should focus on preventing recurrence and restoring rumen epithelial function. Tissue repair may lag behind the normalization of ruminal pH; therefore, grain supply should be restored gradually. The use of partial mixed rations (PMRs) or well-mixed rations can improve dietary uniformity, reduce selective feeding, and stabilize rumen fermentation [178]. To promote rumen epithelial repair and barrier function restoration, butyrate salts or butyrate-yielding precursors can be supplemented to provide energy for rumen epithelial cells, increase their proliferation and repair capabilities, upregulate tight junction protein expression, and restore barrier function [179]. Butyrate and butyrate-yielding precursors should be explicitly considered because butyrate is an important energy substrate and signalling molecule for rumen epithelial cells. It supports epithelial proliferation, papillae development, tight junction regulation, and barrier integrity, and sodium butyrate has been reported to alleviate high-concentrate-diet-induced rumen epithelial barrier damage [72, 180, 181]. Comparative animal and epithelial studies suggest that butyrate-based interventions may support epithelial proliferation and barrier regulation. However, their efficacy, dose, formulation, and timing during recovery from SARA in high-producing dairy cows require direct validation. However, butyrate-based interventions should complement, not replace, correction of the underlying acid-load imbalance, because excessive butyrate exposure under low-pH conditions may also challenge the rumen epithelium [182, 183]. Concurrently, to assess recovery progress and provide early warnings of recurrence risk, continuous rumen pH monitoring and comprehensive multiple indicator alerts should be utilized for periodic health assessments (Table 4). Because epithelial recovery, microbial stabilization, and feeding behaviour may recover at different rates, recurrence monitoring should combine ruminal pH dynamics with feeding behaviour, DMI, milk indicators, inflammatory biomarkers, and clinical signs. In this framework, treatment and recovery are part of the same nutritional management continuum.

**Table 4:** Staged nutritional correction, recovery support, and recurrence prevention following SARA

Treatment stage	Intervention strategy	Expected outcome
Prevention	Continuous monitoring and early warning	Assessment of recovery efficacy; timely detection of recurrence risks
Initial acid-load correction	Dietary structure adjustment	Reduction of ruminal acid load and restoration of conditions supporting stable fermentation
Early rumen stabilization	Support of ruminal buffering capacity	Reduction of postprandial pH depression
Supportive modulation	Yeast-based products or candidate microbial modulators	Support of fermentation stability and modulation of epithelial or inflammatory responses where supported by model-specific evidence
Recovery	Plant extracts	Possible modulation of fermentation patterns
Recovery	Thiamine (Vitamin B_1_)	Support of lactate metabolism under high-grain challenge.
Recovery	Vitamin E	Support of antioxidant capacity and oxidative-stress mitigation
Recovery	PMRs	Stabilization of rumen fermentation; prevention of SARA recurrence

*SARA* Subacute ruminal acidosis, *VFAs* Volatile fatty acids, *LPS* Lipopolysaccharide, *PMRs* Partial mixed rations

## SARA diagnostic and monitoring tools

Accurate SARA assessment should support timely nutritional adjustment rather than provide a diagnostic label alone. For commercial dairy farms, diagnostic tools should be prioritized according to practical applicability, cost, validation status, and whether the result can guide feeding decisions. Routine herd-level indicators remain the first step, whereas biomarkers, omics approaches, imaging methods, and intelligent monitoring systems should be interpreted according to their validation status and practical readiness (Table 5).

**Table 5:** Practical readiness of diagnostic and monitoring tools for SARA assessment in high-producing dairy cows

Category	Tool or indicator	Current practical role	Main limitations	Recommended interpretation
Currently applicable on farm	Ration assessment, starch/RDS supply, peNDF, TMR quality, feed sorting	Routine herd-level risk assessment and nutritional adjustment	Does not diagnose individual SARA; depends on ration accuracy and intake behaviour	Use as first-line risk prevention and management tool
Currently applicable on farm	Milk yield, milk fat percentage, milk fat-to-protein ratio, selected milk fatty acids	Low-cost herd-level screening	Affected by breed, season, lactation stage, dietary fat, and non-SARA diseases	Use with pH, feeding behaviour, and production records
Currently applicable on farm	Feeding behaviour, rumination activity, DMI, sorting behaviour	Early warning of altered intake and rumen function	Non-specific; requires sensor or management records	Use as early alert and to guide feeding management
Currently applicable on farm	Fecal consistency, dung sieving, undigested grain, fecal pH	Practical indicator of hindgut fermentation and digestion	Non-specific; affected by diet, passage rate, infections	Use as supportive on-farm warning tool
Applicable but limited	Repeated or continuous reticuloruminal pH monitoring	Direct acid-load monitoring, especially in research or high-risk groups	Cost, sensor location, calibration, representativeness, interpretation thresholds	Use as core risk indicator, not stand-alone diagnosis
Promising but insufficiently validated	Milk FTIR/milk fatty acid prediction	Noninvasive herd-level prediction	Requires population calibration and validation across farms	Use as screening candidate
Promising but insufficiently validated	LPS, LBP, SAA, Hp, cytokine-related biomarkers	Indicates host inflammatory response	Limited specificity; no unified thresholds; laboratory cost	Use as supportive host-response indicators
Promising but insufficiently validated	Oral microbiota markers	Noninvasive marker-focused screening	Does not fully represent rumen microbiota; markers need cow validation	Use as complementary marker approach
Promising but insufficiently validated	Ultrasound or imaging-based rumen/abdominal assessment	Potential tissue-level evaluation	Operator dependence, limited validation for SARA, cost/time	Use as supportive clinical or research tool

*SARA* Subacute ruminal acidosis, *RDS* Rumen-degradable starch, *peNDF* Physically effective neutral detergent fibre, *TMR* Total mixed ration, *DMI* Dry matter intake, *FTIR* Fourier-transform infrared spectroscopy, *LPS* Lipopolysaccharide, *LBP* Lipopolysaccharide-binding protein, *SAA* Serum amyloid A, *Hp* Haptoglobin

### Routine indicators and on-site evaluation

Routine indicators remain the first step in herd-level SARA assessment because they are inexpensive and closely linked to daily management. However, most of these indicators are indirect and non-specific. Their value lies in identifying herds or groups that require closer evaluation, rather than confirming individual SARA cases. They should therefore be interpreted together with ration structure, feeding behaviour, milk performance, and ruminal pH information where available.

#### Ruminal pH

Ruminal pH remains the most direct indicator of acid-load exposure, but its interpretation depends on sampling method, anatomical site, feeding time, and evaluation criterion. Rumenocentesis, stomach tubing, and indwelling sensors provide different types of information and should not be interpreted using a single universal threshold [8, 114]. Duration below a defined pH threshold, area under the curve, fluctuation amplitude, and postprandial recovery rate are more informative than one-time pH values (Table 6) [80, 189]. In farm practice, pH data should be used as a core risk indicator to guide reassessment of starch/RDS supply, peNDF adequacy, feed sorting, grain processing, and buffering strategy, rather than as a stand-alone diagnostic endpoint.

**Table 6:** Reported ruminal pH criteria used for SARA assessment in dairy cows

Study context	Diet or challenge condition	Measurement approach	Reported pH criterion	Reference
Lactating dairy cows in diagnostic field protocol	Commercial dairy diets	Rumenocentesis	pH ≤ 5.5; herd-level SARA when ≥ 3/12 cows were below the cut-off	[8]
Dairy cows from Australian herds	Pasture- and concentrate-based systems	Rumenocentesis and stomach-tube sampling; cluster analysis with fermentation variables	Acidotic category: mean pH 5.74 ± 0.47; herd classified acidotic when ≥ 3/8 cows were in this category	[184]
Lactating cows in intensive Italian dairy herds	Commercial TMR-based systems	Rumenocentesis	SARA herd: > 33% cows with pH < 5.5; critical risk: > 33% cows with pH < 5.8	[185]
Grazing lactating dairy cows	Perennial ryegrass-based pasture	Rumen fluid pH measurement	SARA: pH ≤ 5.5; marginal: 5.6–5.8; normal: > 5.8	[122]
Lactating cows in German dairy herds	Commercial dairy herds	Rumenocentesis	Individual low pH: ≤ 5.5; herd likely affected when ≥ 3 cows had pH ≤ 5.5	[13]
Lactating dairy cows under experimental SARA induction	Feed restriction followed by grain challenge	Continuous ruminal pH recording	Time and AUC below pH 5.6; time below pH 5.6 increased from 1.10 to 8.26 h/d	[9]
Mid-lactation dairy cows under grain-induced SARA	Partial replacement of TMR with wheat–barley concentrate	Continuous ruminal pH monitoring	Time below pH 5.6; SARA increased time below pH 5.6 from 187 to 309 min/d	[186]
Lactating dairy cows with different acidosis-risk status	Restricted TMR followed by ground barley–wheat grain challenge	Indwelling ruminal pH system	pH < 5.8 and < 5.5; high-risk cows spent 10.6 h/d < 5.8 and 5.9 h/d < 5.5	[187]
Dairy cows under high-concentrate feeding	High-concentrate diet	Ruminal pH monitoring	SARA defined as pH < 5.6 for ≥ 3 h/d	[188]

*SARA* Subacute ruminal acidosis, *TMR* Total mixed ration, *AUC* Area under the curve, *DM* Dry matter

#### Milk composition

Milk yield, milk fat percentage, and milk fat-to-protein ratio are practical herd-level warning indicators because they are routinely recorded in dairy systems [12]. Specific milk fatty acid patterns, including odd- and branched-chain fatty acids and C18 biohydrogenation intermediates, may provide more information on ruminal fermentation disturbances than bulk milk fat percentage. However, the direction and magnitude of these changes vary with diet composition, SARA induction model, and individual susceptibility, and their diagnostic use [190]. Thus, milk indicators are particularly useful as low-cost herd-level screening tools and should be interpreted alongside ruminal pH dynamics, feeding behaviour, and ration evaluation when informing nutritional adjustments.

#### Diarrhoea

Diarrhoea and fecal changes may indicate excessive fermentable substrate escaping the rumen and entering the hindgut. Fecal or dung sieving can detect undigested grain, large fibre particles, foam, mucus, or fibrin casts, which may reflect poor digestion, rapid passage, hindgut fermentation, or epithelial irritation [191–194]. These signs are field-applicable but non-specific, because they can also be affected by infection, ration change, passage rate, and water intake. Their practical value is to prompt checking of starch processing, effective fibre supply, TMR uniformity, and feed sorting rather than to diagnose SARA alone.

#### Clinical assessment

Clinical signs are useful for evaluating the broader health impact of SARA, but they are generally delayed and non-specific. Disorders such as lameness, mastitis, liver dysfunction, or reproductive inflammation may appear after the initial acidotic challenge and can be influenced by many non-SARA factors [13, 36]. Therefore, clinical signs should not be used as primary diagnostic criteria. They are better interpreted as retrospective herd-level evidence that should trigger review of ruminal pH patterns, ration structure, feeding management, and recurrence prevention.

### Diagnostic technologies and biomarkers

Advanced tools may improve early warning and risk classification, but their value depends on whether they can be translated into clear management decisions. Methods that require complex laboratory analysis, high equipment cost, or specialized data processing should be considered supportive rather than routine diagnostic tools. Their practical relevance should be judged by threshold validation, repeatability, cost-effectiveness, and ability to predict response to nutritional intervention.

#### Dietary prediction models and near-infrared spectroscopy

Predictive calculations based on dietary nutritional components are commonly used for early warning of SARA risk. Studies indicated that in a balanced diet, the ratio of peNDF with a length > 1.18 mm to starch content should not be less than 1.00 [195]. Previous research utilized peNDF > 8 mm, starch content (%DM), and DMI (kg/d) to establish the following pH prediction model [5]:

1 $$\mathrm{pH}=5.960-(0.00781\times\mathrm{starch})+(0.03743\times{\mathrm{peNDF}}_{>8mm})-\lbrack0.00061\times({\mathrm{peNDF}}_{>8mm}\times{\mathrm{peNDF}}_{>8mm})\rbrack$$

The prediction model for a rumen pH < 5.8 duration is as follows:

2 $$\text{Time of pH suppression}=124.7+(1.7007\times\mathrm{DMI})+(20.9270\times\mathrm{starch})+(0.2959\times{\mathrm{peNDF}}_{>8mm})-\lbrack0.0437\times(\mathrm{DMI}\times\mathrm{starch}\times{\mathrm{peNDF}}_{>8mm})\rbrack$$

These tools are useful because they are directly connected to nutritional management and can guide preventive adjustment before clinical problems occur. Near-infrared spectroscopy -based grain or diet evaluation may further support rapid assessment of acidogenic potential, but its application depends on robust reference databases linking feed spectra with ruminal fermentation and pH responses [104]. Therefore, dietary prediction and near-infrared spectroscopy are best used as preventive ration-screening tools rather than diagnostic confirmation methods.

#### Reticuloruminal pH boluses

Indwelling wireless pH boluses provide continuous pH data and can identify low-pH duration, postprandial decline, fluctuation amplitude, and recovery patterns [196–198]. Their main limitation is that most boluses are retained in the reticulum or reticuloruminal region, where pH may differ from ventral rumen pH; sensor drift, noise, and location also affect interpretation [4, 28]. Normalized pH-curve indicators may improve detection by reducing the influence of individual variation and sensor drift [199]. In practice, bolus data are most valuable when abnormal pH dynamics are linked to ration reformulation, peNDF adjustment, grain processing control, or feeding-behaviour management.

#### Abdominal ultrasound technology

Abdominal ultrasonography has been explored as a noninvasive method for assessing rumen wall responses to acidotic challenge. Increased rumen wall, mucosal, or submucosal thickness may reflect tissue responses associated with sustained low pH [200, 201]. However, ultrasound is operator-dependent, less directly linked to acid-load dynamics than pH monitoring, and not yet validated as a routine SARA diagnostic method. It should therefore be considered an adjunctive clinical or research tool for evaluating tissue-level effects rather than a primary field-screening approach.

#### Blood and metabolic biomarkers

Blood and metabolic biomarkers can provide supportive information on whether ruminal disturbance is accompanied by systemic inflammatory or metabolic stress. LPS, LBP, SAA, Hp, cortisol, selected cytokine-related responses, ruminal VFAs, lactate, and selected metabolites have been associated with SARA-related fermentation or inflammatory changes [79, 104, 195, 202–205]. However, these indicators are not specific to SARA, because infection, heat stress, other metabolic disorders, and management stressors can produce similar responses. Standardized diagnostic thresholds for commercial dairy herds are also lacking. Therefore, these biomarkers should be interpreted as supportive host-response indicators rather than stand-alone diagnostic criteria, and their practical value depends on integration with pH dynamics, ration assessment, milk performance, feeding behaviour, and clinical observations.

#### Oral microbiota

Buccal or oral swabs are attractive because they are noninvasive and may provide microbial markers related to ruminal acidogenic challenge. In dairy cows, however, buccal swabs do not fully represent the rumen microbial community, although some marker taxa such as *Dialister* spp. may still provide useful information [206]. Comparative evidence from dairy goats supports the marker potential of buccal microbiota, but these markers require validation in high-producing dairy cows before field application [207]. At present, oral microbiota should be regarded as a complementary, marker-focused screening approach. Its field value will depend on whether stable cow-derived markers can be validated across diets, farms, sampling times, and nutritional interventions.

## Future directions

Future SARA research in high-producing dairy cows should place greater emphasis on causal mechanisms rather than descriptive associations. In particular, microbial changes should be interpreted together with microbial functions, metabolite production, epithelial barrier regulation, and host immune responses. Longitudinal studies combining ruminal pH dynamics, microbial activity, epithelial indicators, inflammatory markers, and production responses are needed to clarify which changes initiate SARA progression and which represent downstream consequences or adaptive responses.

Individualized nutrition is another important direction, but it remains dependent on phenotype validation. Cows fed the same ration may differ in eating rate, feed sorting, rumination activity, pH recovery, epithelial absorptive capacity, inflammatory responsiveness, and production loss. Future studies should identify candidate susceptibility phenotypes and test whether cows with different acid-load adaptation capacities respond differently to adjustments in starch/RDS supply, peNDF, grain processing, TMR structure, feeding frequency, buffer use, or yeast-based support.

High-throughput omics and metabolomics may help identify biological signals of SARA and explain individual variation in susceptibility. However, their application should move from broad biomarker discovery toward targeted validation. Candidate microbial, metabolic, or host-response markers need to be evaluated for reproducibility, biological relevance, sampling feasibility, cost, and responsiveness to nutritional correction before they can be used for routine risk assessment.

Bioinformatic models and intelligent monitoring technologies may further support SARA management by integrating pH dynamics, milk records, rumination behaviour, DMI, ration composition, feed sorting, and herd health data. However, these systems should be judged not only by prediction accuracy, but also by interpretability, external validation, cost-effectiveness, and whether they generate actionable feeding recommendations under commercial dairy farm conditions. At present, AI-supported risk classification should be viewed as a future decision-support direction rather than a near-ready diagnostic solution.

## Conclusions

SARA remains a major challenge in high-producing dairy cows because intensive milk production often requires energy-dense diets that increase ruminal acid-load pressure. Rather than being viewed only as a low-ruminal-pH disorder, SARA should be interpreted as a failure of acid-load adaptation across the diet–microbiota–epithelium–host axis. The strongest evidence supports the role of excessive rapidly fermentable carbohydrate supply, especially starch or RDS, together with insufficient physically effective fibre, in disrupting ruminal fermentation stability. Microbial dysbiosis, epithelial barrier impairment, microbial translocation-associated inflammation, milk fat depression, systemic biomarkers, and extra-ruminal responses provide important supportive evidence, but their interpretation should remain context-dependent. Practical management should therefore focus on correcting the underlying acid-load imbalance through coordinated ration formulation, feed delivery, and transition management. Functional additives may provide additional support, but their value depends on the basal diet and should be viewed as complementary tools. Future progress will depend on validating early-warning and decision-support tools under commercial dairy conditions. Reticuloruminal pH dynamics provide the most direct information on acid-load exposure, whereas ration assessment, feeding behaviour, rumination, and routinely collected milk records currently offer greater herd-level practicality. A selective, evidence-based framework may help shift SARA management from delayed recognition of disease consequences toward earlier risk identification, targeted nutritional adjustment, and more stable rumen health and productivity.

## Data Availability

No datasets were generated or analysed during the current study.

## Abbreviations

CD14: Cluster of differentiation 14
DMI: Dry matter intake
HC diet: High-concentrate diet
Hp: Haptoglobin
iE-DAP: γ-D-glutamyl-meso-diaminopimelic acid
LBP: LPS-binding protein
LPS: Lipopolysaccharide
MD-2: Myeloid differentiation factor 2
NFC: Non-fibre carbohydrates
NF-κB: Nuclear factor-kappa B
NOD1: Nucleotide-binding oligomerization domain-containing protein 1
peNDF: Physically effective neutral detergent fibre
pfNDF: Potentially fermentable neutral detergent fibre
PMRs: Partial mixed rations
RDS: Rumen-degradable starch
SAA: Serum amyloid A
SARA: Subacute ruminal acidosis
TLR4: Toll-like receptor 4
TMR: Total mixed ration
VFAs: Volatile fatty acids
SCFP: *Saccharomyces cerevisiae* fermentation products
